# Advancing understanding of the role of IL-22 in myelination: insights from the Cuprizone mouse model

**DOI:** 10.3389/fneur.2024.1411143

**Published:** 2024-07-08

**Authors:** Imen Zamali, Ines Elbini, Raja Rekik, Nour-Elhouda Neili, Wafa Ben Hamouda, Ahlem Ben Hmid, Raoudha Doghri, Mélika Ben Ahmed

**Affiliations:** ^1^Laboratory of Transmission, Control and Immunobiology of Infection, Institut Pasteur de Tunis, Tunis, Tunisia; ^2^Laboratory of Clinical Immunology, Institut Pasteur de Tunis, Tunis, Tunisia; ^3^Faculté de Médecine de Tunis, University of Tunis El Manar, Tunis, Tunisia; ^4^Laboratory of Biomolecules, Venoms and Theranostic Applications (LR20IPT01), Pasteur Institute of Tunis, University of Tunis El Manar, Tunis, Tunisia; ^5^Research Laboratory of Precision Medicine, Personalized Medicine and Oncology Investigation (LR21SP01), Tunis, Tunisia

**Keywords:** IL-22, myelination, myelin basic protein, Cuprizone mouse model, multiple sclerosis

## Abstract

Despite significant advancements in the field, the pathophysiology of multiple sclerosis (MS) remains partially understood, with limited therapeutic options available for this debilitating condition. The precise impact of Interleukin-22 (IL-22) in the context of MS is still incompletely elucidated with some evidence suggesting its protective role. To provide a more comprehensive understanding of the role of IL-22, we investigated its effect on remyelination in a mouse model of demyelination induced by Cuprizone. Mice underwent a 6 week regimen of Cuprizone or vehicle, followed or not by intraperitoneal administration of IL-22. Behavioral assessments including tail suspension and inverted screen tests were conducted, alongside histological, histochemical, and quantitative PCR analyses. In Cuprizone-treated mice, IL-22 significantly improved motor and behavioral performance and robustly promoted remyelination in the *corpus callosum*. Additionally, IL-22 administration led to a significant elevation in MBP transcription in brain biopsies of treated mice. These findings collectively suggest a crucial role for IL-22 in the pathophysiology of MS, particularly in supporting the process of remyelination. These results offer potential avenues for expanding therapeutic strategies for MS treatment. Ongoing experiments aim to further unravel the underlying mechanisms of IL-22 action.

## 1 Introduction

The autoimmune origin of multiple sclerosis (MS) is well established as evidenced by the murine model of experimental auto-immune encephalomyelitis (1). MS lesions emerge upon activation of self-reactive CD4 T cells. The T helper 1 (Th1) population was, for a long time, the sole CD4 lymphocytes incriminated in MS. However, recent findings have linked pro-inflammatory T helper 17 (Th17) cells to MS (2). Yet, the precise mechanisms underlying Th17 involvement in MS pathogenesis remain debated. Along with IL-17A and IL-17F, Th17 cells also produce IL-22, a member of IL-10 family (3). IL-22 plays an important role in the regulation of immunity, inflammation and tissue homeostasis at barrier surfaces. IL-22 is also involved in the process of healing and regeneration of the epithelial barrier during inflammatory episodes (4). Moreover, IL-22 plays a key role in regulating host-protective antimicrobial immunity (5). However, its contribution to the pathology is still controversial. IL-22 has been poorly studied in the context of MS. So far, in combination with IL-17, IL-22 has been suggested to disrupt blood brain barrier (BBB) integrity by breaking tight junctions (6). Yet, IL-22-deficient mice exhibited an unchanged course of EAE compared to wild type mice (7). Moreover, IL-22 Binding Protein (IL-22BP) knock-out mice have an attenuated neuroinflammatory profile of EAE suggesting that IL-22 attenuates disease severity (8). Additional arguments strengthen the rationale of protective effects of IL-22 in MS. IL-22 seems to have pro-survival properties on primary human astrocytes (9) type I interferons modulate astrocyte activity and CNS inflammation via the Aryl hydrocarbon Receptor (AhR) (10). Since IL-22 has been implicated in tissue repair processes in other models of injury and inflammation, we hypothesized that IL-22 could promote remyelination and repair mechanisms, thereby mitigating the effects of demyelination. Herein, we sought to gain insight into the involvement of IL-22 in MS by testing its effect on remyelination in the Cuprizone (CPZ)-induced mouse model.

## 2 Materials and methods

### 2.1 Cuprizone mouse model

Female mice belonging to the C57BL/6 strain were used for the induction of the demyelination model; the mice were bred and provided by the Pasteur institute’s own breeding program. The mouse lineage was initially obtained from Institut Pasteur, Paris, France. The animals, aged from 6 to 8 weeks, were split into 2 groups of 10 and housed in polycarbonate cages with sawdust bedding and maintained in environmentally controlled rooms (22 ± 2°C and 50 ± 10% humidity) with a 12 h light/dark cycle starting at 7 am. In addition, animals underwent routine cage maintenance twice a week, and food and water were available *ad libitum*. All manipulations were performed with the animal’s well-being in mind, excluding all instances of stress and nervousness that might interfere with the results.

Cuprizone [bis-cyclohexanone-oxaldihydrazone] is a low molecular weight copper chelator that induces reversible demyelination in both gray and white matter in the murine brain when consumed orally (11). Cuprizone powder (Sigma-Aldrich, Catalog number C9012) was mixed with corn oil and vortexed to obtain a homogeneous suspension. After a 1 week period of acclimation, mice were fed once a day through oral gavage (using a metallic probe which is inserted directly into the stomach) with a dose equivalent to 300 mg/kg of Cuprizone for a period of 6 weeks. The solution was prepared daily for immediate consumption. A vehicle group was given daily doses of corn oil through oral gavage for the same duration. Following the 6 week period of Cuprizone treatment, behavioral studies were performed, namely the inverted screen test and the tail suspension test. Forty-eight hours later, the vehicle group (CTR-) as well as the positive control group (CTR+) were euthanized and their brains were collected for further testing. The remaining mice were then split into 2 groups; one group would experience spontaneous remyelination (SR) while another group would undergo a treatment phase consisting of an intraperitoneally injection of murine recombinant IL-22 (rIL-22) (E-coli-derived rIL-22, R&D^®^ systems, Catalog Number 582-ML) at 200 mg/kg of animal weight at day 1 and day 5. Ten days after the beginning of rIL-22 treatment, behavioral studies were again performed after the treatment period for comparison purposes. The mice were then euthanized and their brains were collected for further testing.

Sample size calculation was performed through an online calculator^1^ as follow: in order to detect a difference of at least 3 standard deviations in suspension and immobility times and MBP mRNA level between the 2 groups, the number of mice required in each group was 3.

### 2.2 Behavioral studies

#### 2.2.1 The inverted screen test

Invented by Kondziela in 1964, the inverted screen test is used to measure motor strength/coordination of all four limbs. The screen is a 43 cm square of wire mesh comprised of 12 mm squares of 1 mm diameter wire. The screen is then surrounded by a 4 cm deep wooden beading, thus preventing mice from escaping by climbing to the other side (12).

The mouse is first placed in the center of the screen, the latter is then rotated to an inverted position over 2 s, and here the mouse’s head should be the first to decline. The screen is held steadily approximately 30 cm above a padded surface. The test is videotaped until the mouse falls off or until the criterion time of 60 s is reached, in which case the mouse is manually removed from the screen. The recording highlights the precise time in which the mouse fell from the mesh wire screen.

#### 2.2.2 The tail suspension test

The tail-suspension test is a mouse behavioral test useful in assessing manipulations that are expected to affect depression related behaviors. Mice are suspended by their tails with tape, in such a position that it cannot escape or hold on to nearby surfaces. During this test, typically 6 minutes in duration, the resulting escape oriented behaviors are quantified.

This test involves the suspension of mice above the ground by their tails, a measured length of tape is wrapped around the tail of the mouse on one end, approximately 5 mm away from the tip, and the other end is attached to an elevated surface.

The tail suspension test, which typically last for 6 min, requires video recording. Since it involves testing multiple subjects simultaneously, live scoring would be difficult and is not advised. The mouse is considered immobile when all movements and behaviors that are escape related cease and it remains relatively still.

### 2.3 Euthanization and sample collection

Following the experiments, the mice were euthanized through rapid decapitation. The cranium was opened using scissors and the brains of mice that underwent demyelination through Cuprizone treatment and those treated or not by rIL-22 after a 6 week regimen of Cuprizone were collected in a sterile setting.

### 2.4 Histological analysis and Luxol fast blue staining

Removed brains were post-fixed in 10% paraformaldehyde and embedded in paraffin. For light microscopy, 7 μm serial coronal paraffin sections between bregma −0.82 mm and bregma −1.94 mm according to Paxinos Franklin Mouse Brain Atlas were cut with a rotary microtome (RM2245, Leica, Wetzlar, Germany) and evaluated microscopically.

Brain sections of mice from different treatment groups were stained using Luxol Fast Blue (LFB, Sigma-Aldrich, Catalog number L0294) in order to determine the level of demyelination. Fast luxol blue is a sulfonated copper phthalocyanine dye. This dye is soluble in alcohol and is attracted to the bases contained in the phospholipids of myelin. Excess dye is removed with 95% ethanol and then the tissue section is differentiated with lithium carbonate and 70% ethanol until the gray matter is colorless. With LFB staining, the myelin is stained blue-green and the neurons are stained purple.

### 2.5 Image acquisition and analysis

The imaging process involved the utilization of an Olympus BX51 microscope equipped with an Olympus DP21 color camera, capturing images at 10×, 40× and 100× magnifications. Consistency in microscope and camera parameters, including light level, exposure, and gain, was maintained across all acquired images. Image Pro Plus (v6.2) software facilitated image capture. For standardized assessment, circular regions of interest (ROIs) of predetermined dimensions were manually positioned at the center of microphotographs depicting anatomically matched LFB-stained sections, specifically within the corpus callosum. Myelin densities within LFB-stained images were quantified by measuring the mean intensity of the red channel (IR) within the designated ROI (13) by Qu-Path 0.3.1 software. Image analysis was performed utilizing the ImageJ software, thereby ensuring consistent implementation of procedures across all images. The images were initially converted to 8-bit, and demyelination areas were delineated using threshold algorithms. Notably, the Otsu threshold algorithm was employed to identify demyelinated regions that were positively marked (14).

### 2.6 Immuno-histochemistry for MBP expression

For the immunostaining procedure, brain sections were first deparaffinized and rehydrated. To block non-specific binding, the samples were incubated for 1 h in a solution of 0.1% bovine serum albumin (BSA) (Sigma) in 0.1% Triton X-100/PBS. Subsequently, the sections were incubated overnight with a mouse anti-MBP primary antibody (14,000, Sigma, Cat # MAB42282). After primary antibody incubation, the sections were treated with a horseradish peroxidase (HRP)-conjugated secondary antibody (R&D systems, Cat # VC002).

### 2.7 MBP mRNA expression

After mechanical disruption (FastPrep-24^™^ 5G Instrument), total RNA was extracted from cryopreserved brain biopsies employing the RNeasy Mini Kit (Qiagen, Catalog number 74104) following the manufacturer’s recommended protocol. Subsequently, the isolated RNA underwent reverse transcription using Murine-Moloney Leukemia Virus (MMLV) reverse transcriptase and random hexamers (Promega), adhering to standard procedures. The quantification of MBP mRNA was conducted through real-time PCR, using an available gene expression assay^®^ (Mm99999915_g1, Cat # 4331182) and Taqman PCR Master Mix (Cat # 4304437) from Thermo Fisher Scientific. The endogenous gene Ribosomal Protein, Large, PO (RPLPO) was used as a reference gene and the results were calculated using the 2^−ΔCT^ method, with ΔCT representing the difference in threshold cycles between MBP and reference genes.

### 2.8 Statistical analyses

Data were compared using Statview and GraphPad Prism softwares. Correlations were calculated with Spearman’s Rank correlation coefficient. In the animal model, the results were expressed as the mean of the values of each group and the standard error of the mean (SEM). Comparisons between groups were conducted by one-way analysis of variance (ANOVA) followed by Tukey’s and Holm Sidak post-hoc test after performing certain verifications (Skewness less than 2 and Kurtosis was less than 9 in absolute value). Differences with *p* < 0.05 were considered statistically significant; *p < 0.05, **p < 0.01, and ***p < 0.001.

## 3 Results

To test the effect of IL-22 on myelination, several experiments (*n* = 3) were conducted with a total of 24 mice (Supplementary Figure S1). In each experiment, 8 mice were tested. Two mice were given daily doses of corn oil via oral gavage, serving as the vehicle control group (CTR-). The remaining mice were fed Cuprizone. After 6 weeks, behavioral tests were performed on vehicle and Cuprizone groups, and two mice from each group were euthanized for brain testing, corresponding to the CTR- and CTR+ groups, respectively.

The remaining mice were divided into two groups: two mice were treated with rIL-22, and two mice underwent spontaneous remyelination (SR). Behavioral tests were conducted on the last two groups after a period of 10 days. At the end of the experiment, all mice were euthanized for brain analyses (Supplementary Figure S1).

**Figure 1:**
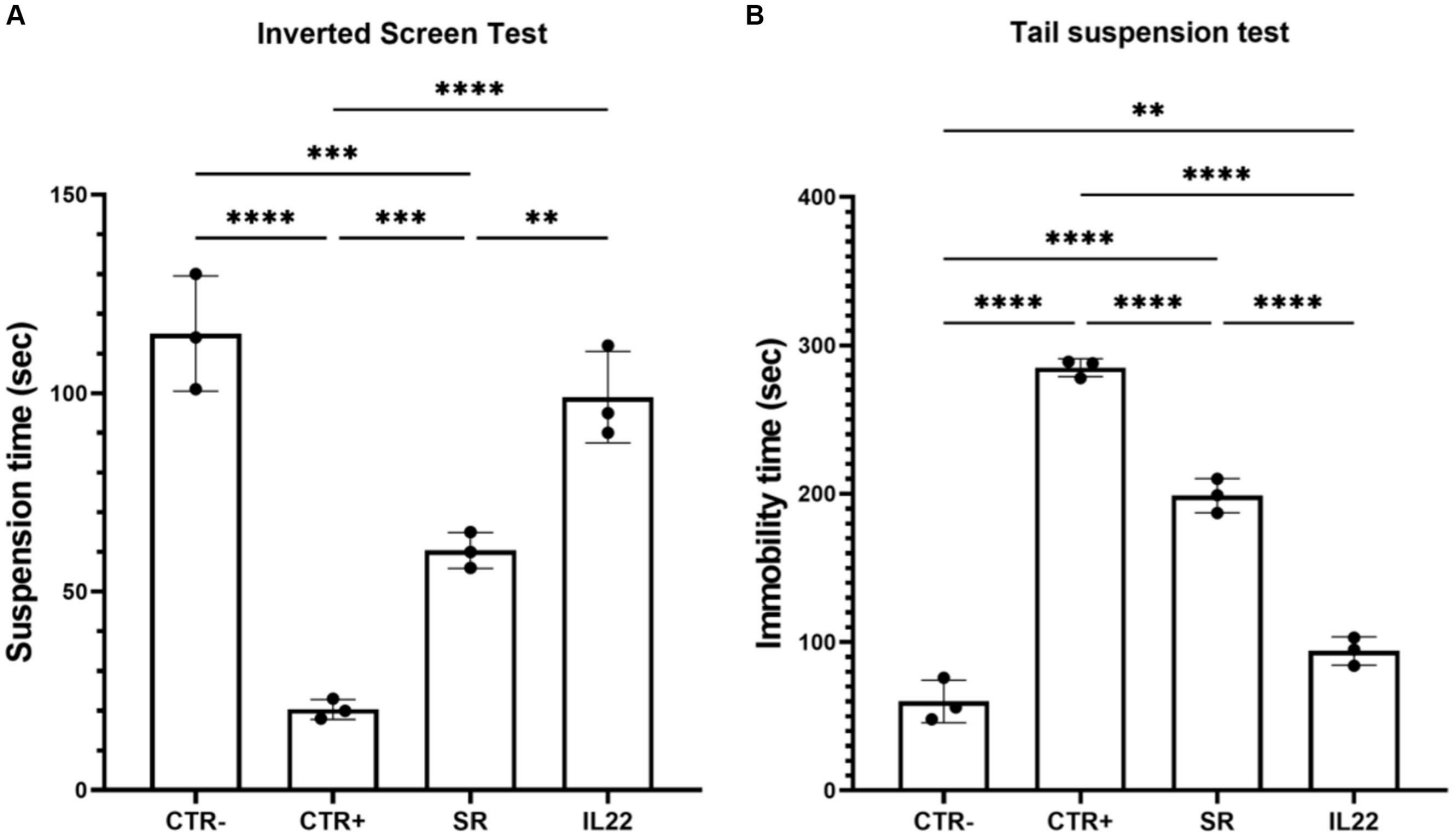
Behavioral tests in studied mice. Mice were treated 6 weeks with Cuprizone or vehicle (*n* = 8). Cuprizone-treated mice were then injected or not (spontaneous remission group or SR group) intraperitoneally by rIL-22 (200 mg/kg of animal weight) at day 1 and day 5 (*n* = 3 for each group). Kondziela inverted screen test **(A)** and tail test **(B)** were performed in the vehicle group (CTR-) and the treated-mice (CTR+) before or after the injection with rIL-22. Results are expressed as the time in seconds at which the mouse fall **(A)** and the immobility time for the tail text **(B)**. Bars indicate mean with standard deviations *p < 0.05, **p < 0.01, ***p < 0.001, and ****p < 0.0001. The results are from three representative mice. The dots represent the result from each tested mouse. Comparisons between groups were conducted by one-way analysis of variance (ANOVA) followed by Tukey’s and Holm Sidak post-hoc test.

Regarding the behavior tests, the Kondziela inverted screen and tail tests revealed a notable positive impact of the IL-22-treated group in contrast to mice undergoing SR (*p* = 0.04) (Figures 1A,B).

**Figure 2:**
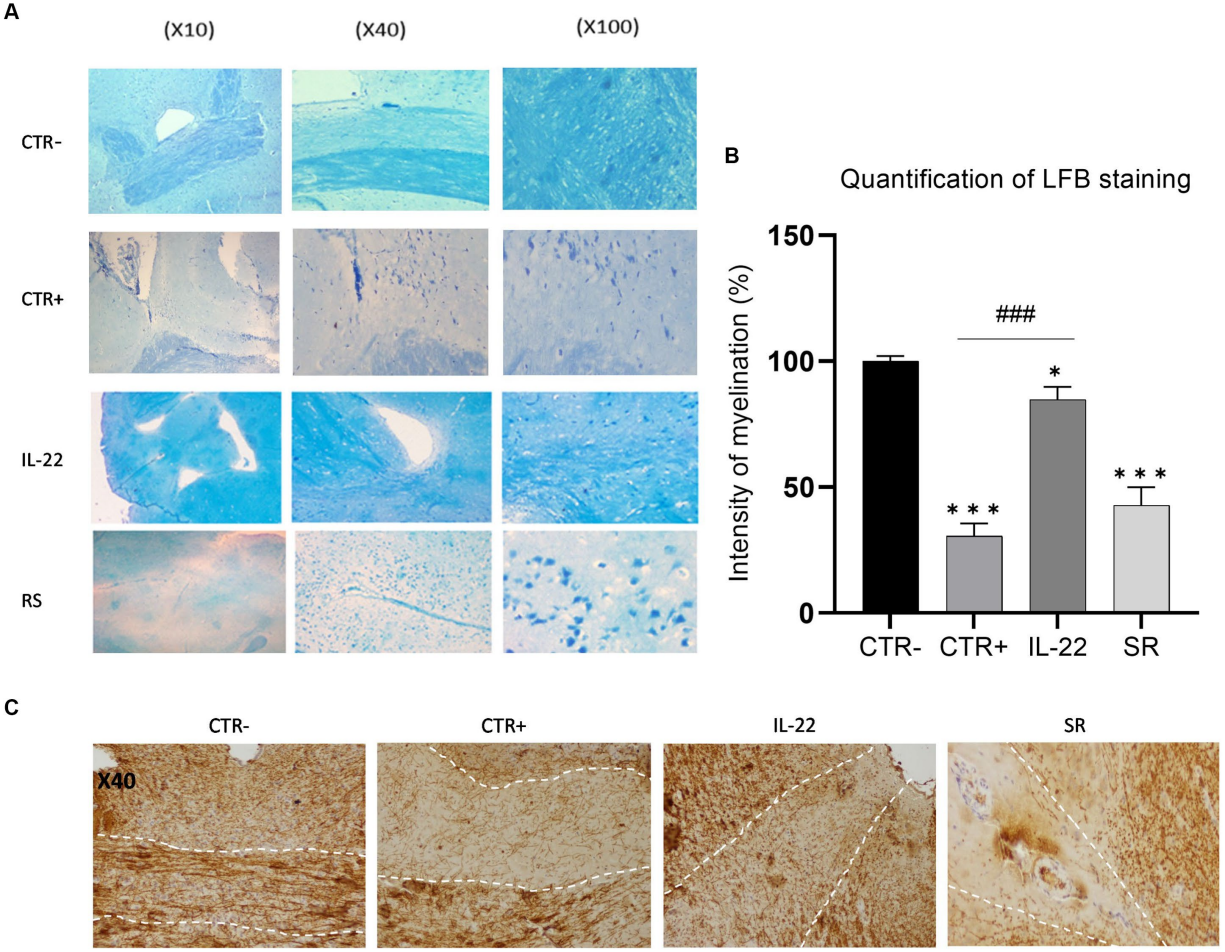
MBP expression in brain’s mice after rIL-22 treatment. Mice were treated 6 weeks with Cuprizone or vehicle. The vehicle group (CTR-) as well as a sub-group of the Cuprizone-treated group (CTR+) were euthanized and their brains were collected for testing. The remaining Cuprizone-treated mice were injected or not (Spontaneous remission group or SR group) intraperitoneally by rIL-22 (200 mg/kg) at day 1 and day 5 (*n* = 3). After 10 days, the treated mice were euthanized and their brains were collected for testing. **(A)** Corpus Callosum sections from different treatment groups were stained using Luxol Fast Blue. Results of one representative mouse from each group are shown. **(B)** Quantification of the LFB staining was evaluated by Qu-Path 0.3.1 and image J softwares according to the staining of CTR- (100%). **(C)** Corpus Callosum sections from different treatment groups were immune-stained using anti-MBP antibody. Results of one representative mouse from each group are shown. * Significance when compared to CTR-, # significance when compared to CTR+. Comparisons between groups were conducted by one-way analysis of variance (ANOVA) followed by Tukey’s and Holm Sidak post-hoc test.

Following the behavioral tests, mice were euthanized, and their brains were collected for staining with Luxol Fast Blue. Analysis of the *Corpus callosum* staining was conducted, and scores were assigned based on myelin basic protein-stained images to evaluate myelination levels. Our data demonstrated a moderate level of remyelination in mice 10 days after the cessation of Cuprizone treatment (spontaneous remyelination group) (Figures 2A,B). Remarkably, rIL-22-treated mice exhibited a markedly enhanced myelin staining similar to that of the control group (vehicle-treated), suggesting a potent induction of myelination by IL-22. Immuno-histochemistry using anti-MBP antibody showed similar results (Figure 2C).

**Figure 3:**
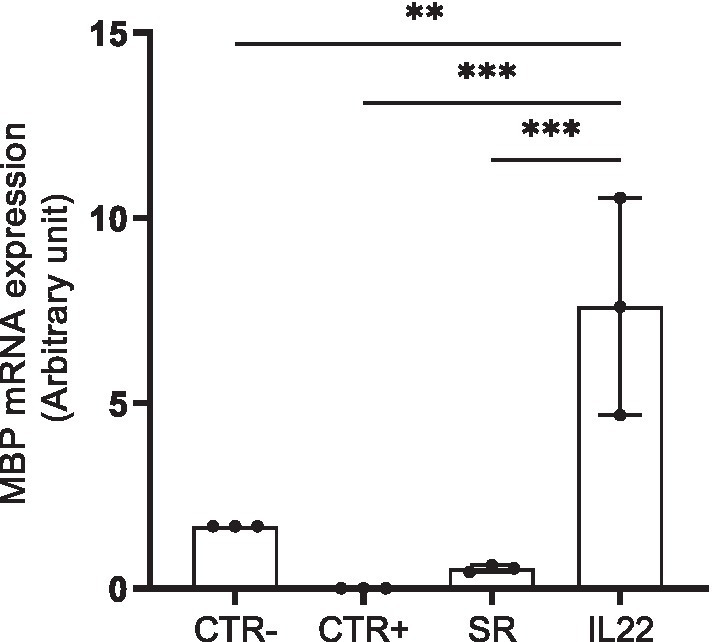
MBP mRNA expression in mouse brain biopsies. mRNA expression of MBP was evaluated by RT-quantitative PCR in cryopreserved brains collected from the vehicle (CTR-), Cuprizone-treated (CTR+) groups as well as spontaneous remission (SR), IL-22-treated groups. Bars indicate mean with standard deviations **p < 0.01 and ***p < 0.001. Comparisons between groups were conducted by one-way analysis of variance (ANOVA) followed by Tukey’s and Holm Sidak post-hoc test. The results are from three representative mice. The dots represent the result from each tested mouse.

Finally, MBP mRNA gene expression was assessed in the cryopreserved brain biopsies collected from all tested groups. As shown in Figure 3, Cuprizone-treated mice (CTR+ group) showed a down-modulation of MBP mRNA expression compared to CTR- group. In contrast, mice treated with rIL-22 exhibited a robust and statically significant upregulation of MBP expression, surpassing not only that of the spontaneous remission group by 14 times but also exceeding the MBP mRNA expression in the CTR- group.

## 4 Discussion

The abundance of immune cells such as T cells and their products in CNS lesions of MS patients strongly support the concept that MS is a dysimmune disease advocating for an autoimmune etiology. The autoimmune origin of MS is well established as evidenced by the murine model of extrinsic autoimmune encephalomyelitis (EAE). The T helper 1 (Th1) and T helper 17 (Th17) described as pro-inflammatory cells, have been involved in MS (15). Recent attention has been focused on novel T lymphocyte subpopulation identified as IL-22 producing Th22 lymphocytes. Some findings seem to involve IL-22 in the inflammatory process localized within the demyelinating lesion itself or in that of the blood–brain barrier (BBB) resulting in increasing permeability to lymphocyte migration (6). Conversely, other data suggest a protective role of Th22 cells during MS by inhibiting, among others, the differentiation of pathogenic Th17 cells. Some experimental data corroborate this hypothesis by showing, for example, that TCDD (2,3,7,8-tetrachlorodibenzo-p-dioxin), a toxic synthetic ligand of the Aryl Hydrocarbon Receptor (AhR)—a transcription factor of IL-22—induces an inhibition of the development of EAE symptoms in mice (16). This effect is attributed not only to the development of a Th22 population at the expense of the Th17 population but also through the expansion of CD4+ CD25+ FoxP3+ regulatory T cells. Interestingly, another non-toxic AhR ligand, ITE for 2-(1′H-indole-3′-carbonyl)-thiazole-4-carboxylic acid methyl ester, has similarly demonstrated efficacy in attenuating EAE symptoms in mice (17). Collectively, these findings imply a protective role for IL-22 in MS and suggest that AhR ligands hold promise as potential candidates for therapeutic interventions in this condition.

Our data suggest a pivotal role of IL-22 in the pathophysiology of MS, specifically in sustaining the process of remyelination. The involvement of IL-22 in the myelination process was assessed through *in vivo* experiments by using a murine demyelination model (Cuprizone mouse model). This model is widely used to study demyelination and remyelination processes, which are central to understanding MS pathology and potential therapeutic interventions. Cuprizone mouse model serves as a toxic-induced demyelination model based on oral intoxication with the copper-chelator Cuprizone. This induces oligodendrocyte apoptosis within a short timeframe, closely followed by the activation of innate immune cells in the brain, specifically astrocytes and microglia, ultimately culminating in demyelination across white and grey matter brain areas. In our study, we administered Cuprizone, a neurotoxic agent, to female mice of C57BL/6 J strain for a period of 6 weeks. Subsequently, rIL-22 was administered to demyelinated mice by intraperitoneal (IP) on days 1 and 5. Behavioral, histological, histochemical and quantitative PCR analyses were conducted in this study after a 10 day period allowing time for spontaneous remyelination. Results from both behavioral tests revealed that rIL-22 significantly restored motor performance in Cuprizone-intoxicated mice, surpassing the effects observed during spontaneous remyelination. Concurrently, histopathological examination using a specific dye, LFB, demonstrated enhanced myelination in the brains of rIL-22-treated mice compared to untreated counterparts. Interestingly, induction of MBP expression was associated with significant induction of MBP gene transcription. Our findings provide compelling evidence for the impact of IL-22 on remyelination, representing the first demonstration of such an effect.

Notably, the positive impact of IL-22 on myelination may arise either directly through its effect on oligodendrocytes or through the modulation of mediators produced by astrocytes. The interaction between oligodendrocytes and astrocytes stands as a pivotal stage in myelin biosynthesis (18). Astrocytes play a dual role by secreting a diverse array of factors that can either enhance, i.e., CNTF (ciliary neutrotrophic factor) and FGF-2 (fibroblast growth factor-2) or impede, i.e., CXCL10 (C-X-C motif chemokine 10) this crucial neurological process (18, 19). Moreover, throughout CNS development, astrocytes play a vital role in supporting oligodendrocytes metabolically, providing essential nutrients, lipids (20–22). They also produce neurotropic and growth factors as well as neuropoietic cytokines such as Brain-derived neurotrophic factor (BDNF) or Heparin-binding EGF-like growth factor (HB-EGF) that exert reparative and tissue-protective functions after acute inflammatory lesions or in neurodegenerative disorders (23). Interestingly, IL-22 has been shown to induce the expression of several neuroprotective factors that could potentially enhance the survival and function of oligodendrocytes in the Cuprizone mouse model. *In vitro* experiments are ongoing to decipher the mechanisms underlying the effect of IL-22. The apparent contradiction between our results and the lack of effect observed in the IL-22 knockout model of EAE (7) suggests complexity in the role of IL-22 in MS. It is conceivable that IL-22 influence becomes more apparent during the later stages, particularly during the remyelination phase following the acute phase of EAE thus explaining such a negative result with the IL-22 knockout model. More recently, Eken et al. confirmed that IL-22 knockout has no effect on the progression of MOG35-55 peptide-induced murine EAE. However, they demonstrated that the temporal overexpression of murine IL-22 provides protective effects, evidenced by reduced EAE disease scores and decreased demyelination, thus supporting our findings (24). Moreover, they noticed a reduced infiltration of IL-17+ lymphocytes in the CNS, pointing to additional protective effects of IL-22.

Our study has several strengths. It focused on the effect of IL-22 on myelination what has never been addressed before. The effect on myelination was, furthermore, tested through various approaches, including either behavioral or biological assessments. The major limitation of our work is the lack of analysis of the immune cell changes or the expression of neuroprotective molecules that could be involved in this effect. This should be performed in the continuation of our work.

In conclusion, our findings constitute the first step towards delineating the therapeutic potential of IL-22 in the management of patients with multiple sclerosis (MS). Yet, extensive research remains imperative including more in-depth investigation using both cellular and animal, to deepen the pre-clinical study before any impact on patients could be considered. Furthermore, a comprehensive understanding of the molecular mechanisms underlying CNS myelination and oligodendrocyte differentiation is crucial for advancing therapeutic strategies for MS.

## Data availability statement

The original contributions presented in the study are included in the article/Supplementary material, further inquiries can be directed to the corresponding author.

## Ethics statement

The animal study was approved by the Biomedical Ethics Committee of the Pasteur Institute of Tunis (No. 2022/3/I). The study was conducted in accordance with the local legislation and institutional requirements.

## Author contributions

IZ: Conceptualization, Formal analysis, Investigation, Methodology, Writing – original draft, Writing – review & editing. IE: Formal analysis, Writing – review & editing, Investigation, Methodology. RR: Formal analysis, Writing – review & editing, Supervision. N-EN: Formal analysis, Writing – review & editing, Investigation. AB: Formal analysis, Writing – review & editing. RD: Formal analysis, Writing – review & editing. MB: Conceptualization, Formal analysis, Investigation, Methodology, Supervision, Writing – original draft, Writing – review & editing. WB: Formal analysis, Writing – review & editing.

## Footnotes

^1^ https://statulator.com/SampleSize/ss2M.html
