# Supplementary figures and images for: Advancing understanding of the role of IL-22 in myelination: insights from the Cuprizone mouse model
Source: Front Neurol. 2024 Jul 8;15:1411143. doi: 10.3389/fneur.2024.1411143 (PMC11260746; doi:10.3389/fneur.2024.1411143)

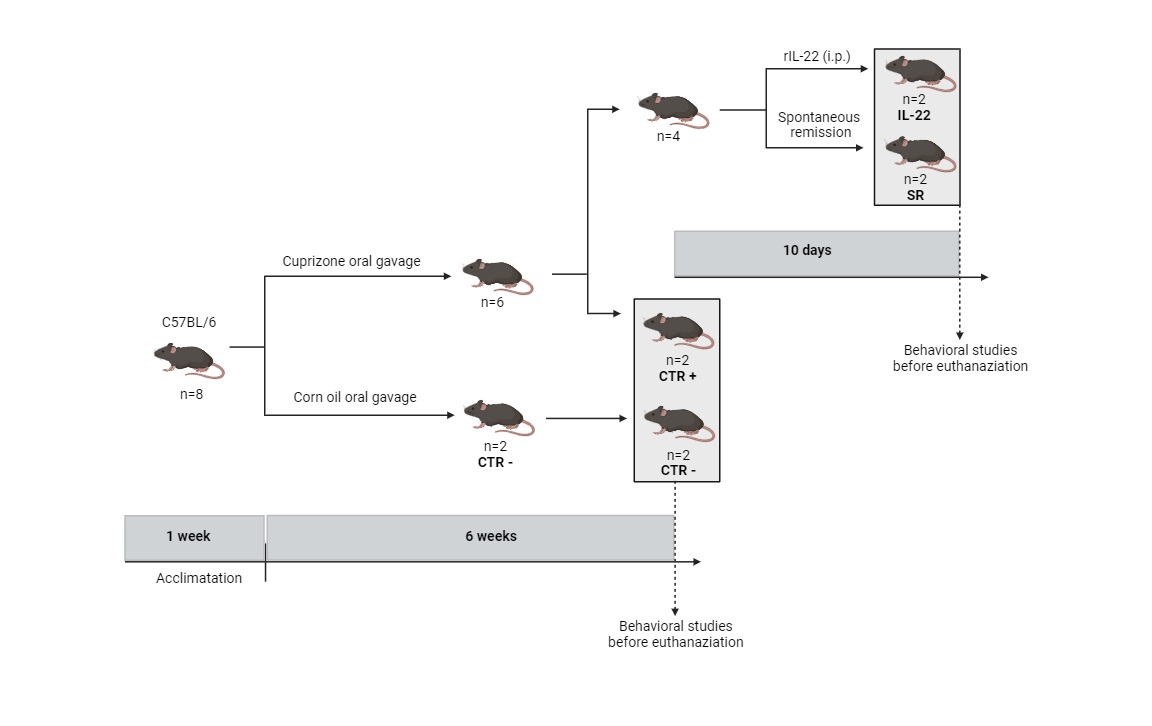

Supplement: Supplementary file 1 [file Image_1.JPEG]
